# Strontium isotope ratios of human hair record intra-city variations in tap water source

**DOI:** 10.1038/s41598-018-21359-0

**Published:** 2018-02-20

**Authors:** Brett J. Tipple, Luciano O. Valenzuela, James R. Ehleringer

**Affiliations:** 1 Department of Biology, University of Utah, Salt Lake City, UT 84112 USA; 2 Global Change and Sustainability Center, University of Utah, Salt Lake City, UT 84112 USA; 3 CONICET, Laboratorio de Ecología Evolutiva Humana, UNCPBA-Quequén, Buenos Aires, Argentina

## Abstract

The oxygen (^18^O/^16^O) isotope analysis of hair is commonly applied to reconstruct an individual’s residence history. However, region-of-origin as determined from oxygen isotope values (*δ*^18^O) alone is often spatially indistinct. Adding additional geochemical recorders can refine region-of-origin estimates. In this capacity, strontium (^87^Sr/^86^Sr) isotope analysis has attracted increased interest. While ^87^Sr/^86^Sr reflects the influences of local geology, ^87^Sr/^86^Sr of hair includes both external environmental signals as well as the internal dietary indicators. To better understand the impact of these contributions to the spatial signal encoded within ^87^Sr/^86^Sr of hair, human hair was collected from three locations within Salt Lake City, Utah along with the donor’s sex. The ^87^Sr/^86^Sr and *δ*^18^O of hair and local tap water were measured. There were no significant relationships between sex and either *δ*^18^O or ^87^Sr/^86^Sr of hair, nor between collection location and the *δ*^18^O of hair. However, we found significant associations between collection location and ^87^Sr/^86^Sr of hair. These findings suggest that interactions with local water may be an important source of Sr to human hair and that the ^87^Sr/^86^Sr of hair may have the capacity to record differences in ^87^Sr/^86^Sr of tap waters on small spatial scales.

## Introduction

The stable oxygen (^18^O/^16^O) isotope values (*δ*^18^O) of human tissues can be utilized to identify and reconstruct an individual’s region-of-residence or origin as *δ*^18^O of human tissue relates largely to the *δ*^18^O of an individual’s drinking water, which varies with geography^1^. Numerous studies have applied the *δ*^18^O of human tissues to determine the likely origin of modern, historic, and prehistoric humans^2–9^. However, predictions of origin using *δ*^18^O can be geographically broad^10,11^. Similar to *δ*^18^O, variations in the strontium (Sr) isotope ratios (^87^Sr/^86^Sr) relate to geography and the analysis of ^87^Sr/^86^Sr in human tissues has been utilized to address many historical^6,12–14^ and prehistorical human provenance questions^14–18^. With ^87^Sr/^86^Sr, the age and geology of the underlying bedrock control the ^87^Sr/^86^Sr in soils, waters, plants, and animals^19–21^, which are incorporated into human tissues^22^. Thus, combining the independent *δ*^18^O and ^87^Sr/^86^Sr systems may allow for greater resolution in human provenance predictions.

Measurements of both *δ*^18^O and ^87^Sr/^86^Sr in tooth enamel have been successfully applied in several modern^23,24^ and prehistorical applications^25^ to identify the probable locations where an individual spent their early life. The measurement of *δ*^18^O and ^87^Sr/^86^Sr in hydroxylapatite tissues from humans is straightforward with tooth enamel having an average of 544 μg g^−1^ Sr^26^. Where tooth enamel represents early periods in an individual’s life, the isotope values of hair keratin represent much more recent intervals of time. Thus, the combined measurement of *δ*^18^O and ^87^Sr/^86^Sr in hair is emerging as a very attractive approach to understand an individual’s very recent life history^27^. This combination of isotopes has many potential applications, especially recognizing known variations in tap waters that exist within and among metropolitan regions^28–30^. However, while the *δ*^18^O systematics in keratin is relatively well understood and commonly applied^1^, the Sr isotopic system in human hair is not^22^.

The various means of Sr incorporation into internal human tissues are ingestion of food and beverages in addition to inhalation of aerosols and particles^31,32^; however, Sr in hair – an external tissue – represents a complex mixture of both these endogenous and exogenous sources of Sr^33^. Endogenous sources of Sr to hair originate from the body’s Sr pools within blood and bones^31,32^, while exogenous sources represent external environmental influences from aerosols, particulates, and environmental waters^27,33,34^. The overwhelming importance of exogenous contributions is evidenced by elevated Sr concentrations in the cortex of the hair^35,36^ as well as continual increases in Sr concentration from the proximal to distal terminus of the hair^27,37^. Chemical separation and isolation of the exterior and interior Sr pools demonstrated that these inputs may impart unique ^87^Sr/^86^Sr ratios and bias the overall ^87^Sr/^86^Sr value^34^. Distinguishing the impact of these two potentially conflicting Sr sources on the overall ^87^Sr/^86^Sr value is required to fully utilize ^87^Sr/^86^Sr of hair for the reconstruction of region-of-residence and remains a fundamental question in the field.

To begin to distinguish the importance of endogenous and exogenous Sr to hair and create a better understanding of how these sources of Sr relate to the spatial signal encoded within ^87^Sr/^86^Sr of hair, we collected and analyzed the ^87^Sr/^86^Sr and *δ*^18^O of human hair from three locations within a single city (Salt Lake City, Utah, USA). In addition to ^87^Sr/^86^Sr and *δ*^18^O, the sex of the hair donors were also gathered, as well as, the ^87^Sr/^86^Sr and *δ*^18^O of tap waters collected from six sites near hair collection locations. Our aim was to explore the relationships of ^87^Sr/^86^Sr and *δ*^18^O of hair at the city-scale and to begin to develop a better understanding of the geospatial signal encoded in the ^87^Sr/^86^Sr of hair.

## Results

### Oxygen isotope values of hair

**Figure 1:**
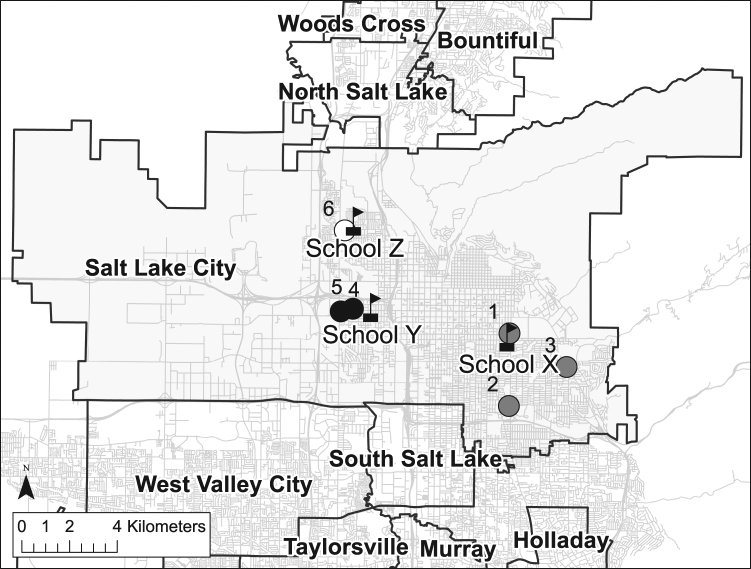
Location map showing the collection locations of hair and tap water. Salt Lake City is highlighted. School locations are shown with flagged symbols and tap water collection locations are shown with points. Point shading indicates groupings with group A sites shown in grey, group B sites shown in black, and the group C site shown in white. Figure produced using ArcGIS 10.4 (ESRI; Redlands, CA, USA).

**Figure 2:**
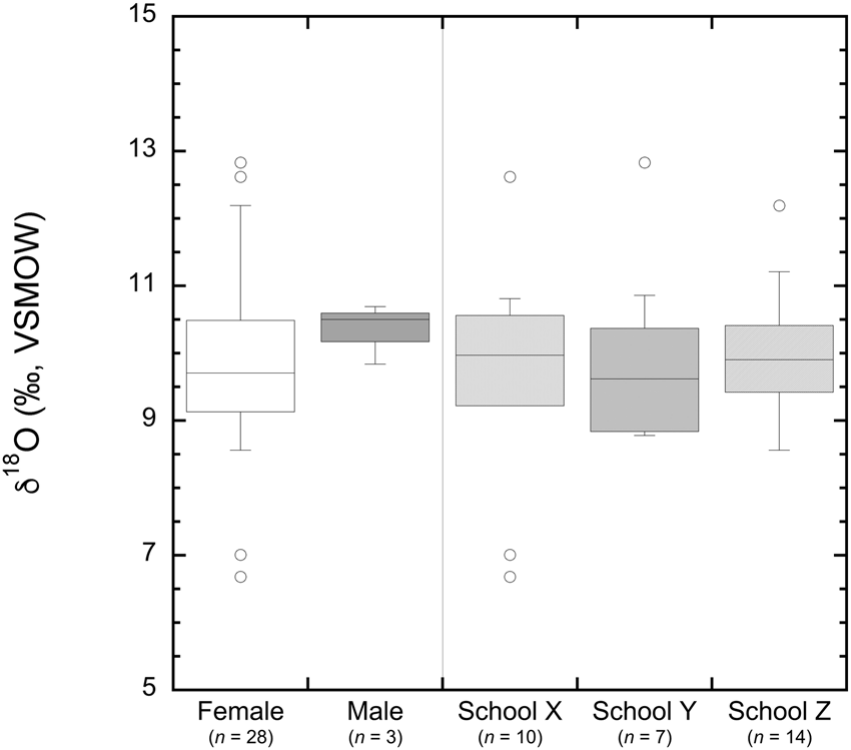
Box and whisker plot showing the *δ*^18^O values of hair from female and male donors and hair data isolated by collection location.

Sixty-seven hair samples were obtained from students from three public schools (Fig. 1). We analyzed thirty-one hair samples for *δ*^18^O and found hair samples ranged in *δ*^18^O from 6.7 to 12.8‰ with a mean *δ*^18^O of 9.9 ± 0.8‰ (1*σ*, *n* = 31). The mean *δ*^18^O of hair samples from females was 9.8 ± 1.4‰ (1*σ*, *n* = 28), while the mean *δ*^18^O of hair samples from males was 10.3 ± 0.4‰ (1*σ*, *n* = 3) (Fig. 2). While our dataset was weighed toward female participants, the *δ*^18^O of hair from females was not significantly different than *δ*^18^O of hair from males (Student’s *t-*test, *p* = 0.5370). When grouped by collection location, we found the mean *δ*^18^O of hair samples from individuals at Schools X, Y, and Z were 9.7 ± 1.8‰ (1*σ*, *n* = 10), 10.0 ± 1.5‰ (1*σ*, *n* = 7), and 10.0 ± 0.9‰ (1*σ*, *n* = 14), respectively (Fig. 2). Here, the *δ*^18^O of hair from individual schools were not significantly different from one another (one-way ANOVA, *p* = 0.8438).

### Oxygen isotope values of water

**Figure 3:**
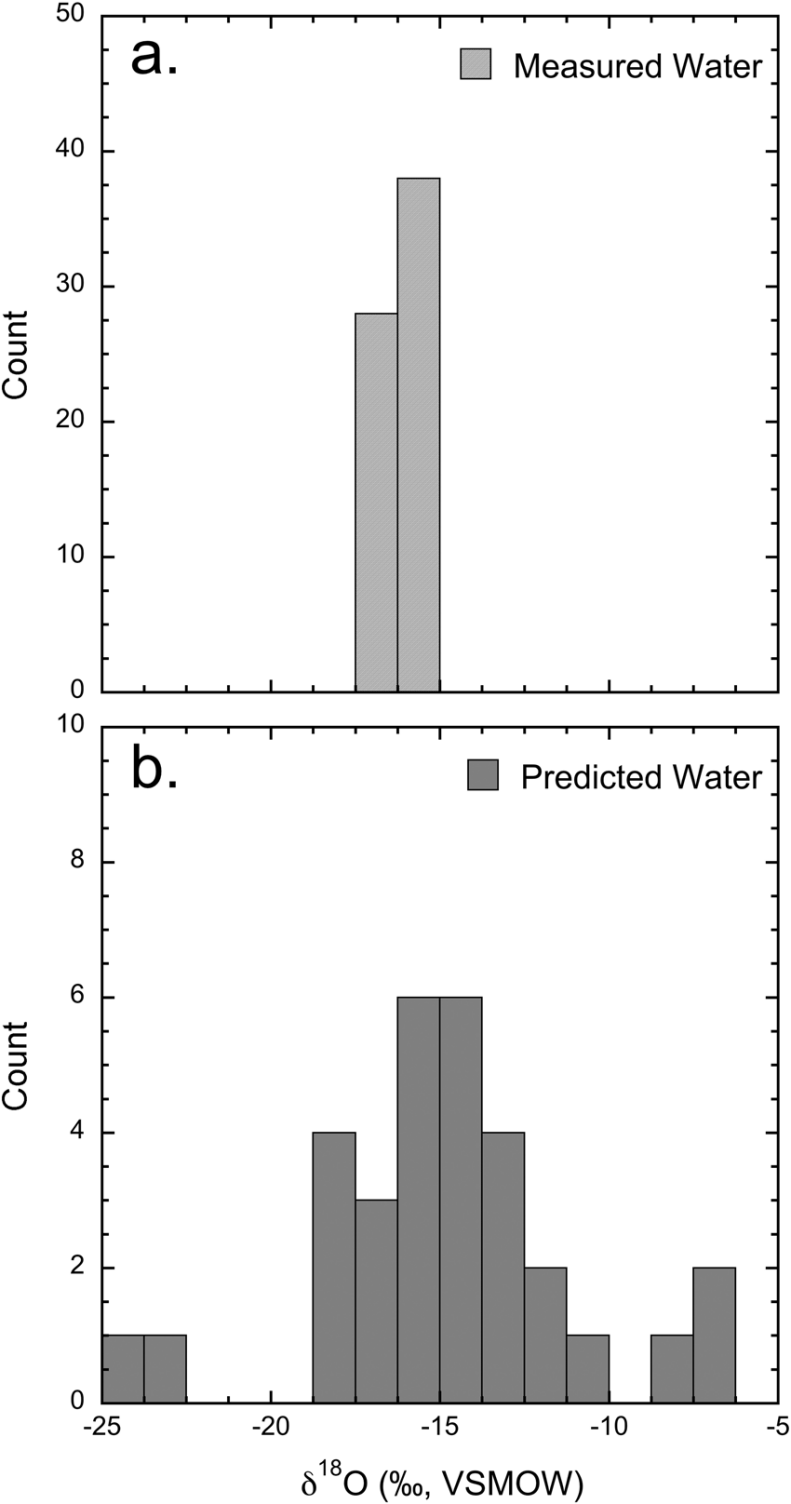
Histogram showing the measured *δ*^18^O values of tap water at collection locations and the predicted *δ*^18^O values of drinking water from the measured *δ*^18^O values of hair.

Water samples were collected from taps in six public buildings four times a year for four years (Fig. 1). The mean *δ*^18^O of collected tap water was −16.3 ± 0.2‰ (1*σ*, *n* = 66) with a range from −15.3 to −16.8‰ (Table 1). Following Ehleringer *et al*.^1^, the *δ*^18^O of drinking water predicted from measured *δ*^18^O of hair ranged between −12.5‰ to −17.0‰ with a mean *δ*^18^O of −14.8 ± 3.7‰ (1*σ*, *n* = 31) (Fig. 3). Here, we found the measured *δ*^18^O of tap water was not significantly different than *δ*^18^O of drinking water predicted from hair (Welch’s *t-*test, *p* = 0.0707).

**Table 1:** Oxygen and strontium isotope ratios of tap waters from sites within Salt Lake City, Utah.

Site	Latitude	Longitude	*δ*^18^O (‰, VSMOW)				^87^Sr/^86^Sr			
			Mean	Min	Max	*n*	Mean	Min	Max	*n*
**1**	40.7523	−111.8544	−16.1 ± 0.3	−16.4	−15.6	8	0.71036 ± 0.00026	0.71002	0.71076	8
**2**	40.7249	−111.8543	−16.0 ± 0.4	−16.8	−15.3	12	0.71032 ± 0.00023	0.70999	0.71071	11
**3**	40.7398	−111.8259	−15.9 ± 0.4	−16.6	−15.3	11	0.71018 ± 0.00032	0.70949	0.71060	10
**4**	40.7599	−111.9382	−16.1 ± 0.4	−16.5	−15.5	13	0.71190 ± 0.00180	0.70908	0.71434	12
**5**	40.7610	−111.9322	−16.0 ± 0.4	−16.8	−15.4	9	0.71196 ± 0.00196	0.70906	0.71493	8
**6**	40.7905	−111.9366	−16.2 ± 0.4	−16.7	−15.3	13	0.71014 ± 0.00060	0.70919	0.71132	12

### Strontium abundance of hair

Sixty-seven hair samples were analyzed for Sr elemental abundance [Sr]. We found [Sr] ranged from 0.08–27.5 μg g^−1^, while the mean [Sr] of hair samples from female and male participants was 7.8 ± 5.0 μg g^−1^ (1*σ*, *n* = 60) and 3.7 ± 3.5 μg g^−1^ (1*σ*, *n* = 7), respectively. [Sr] of hair samples from female participants was significantly higher than [Sr] of hair from males (Welch’s *t-*test, *p* = 0.0003). The mean [Sr] of hair samples from students at Schools X, Y, and Z were 7.4 ± 4.7 μg g^−1^ (1*σ*, *n* = 11), 8.1 ± 4.8 μg g^−1^ (1*σ*, *n* = 29), and 6.7 ± 4.0 μg g^−1^ (1*σ*, *n* = 27), respectively. We did not find any significant differences in [Sr] of hair samples from collection locations (one-way ANOVA, *p* = 0.5510).

### Strontium isotope ratios of hair

**Figure 4:**
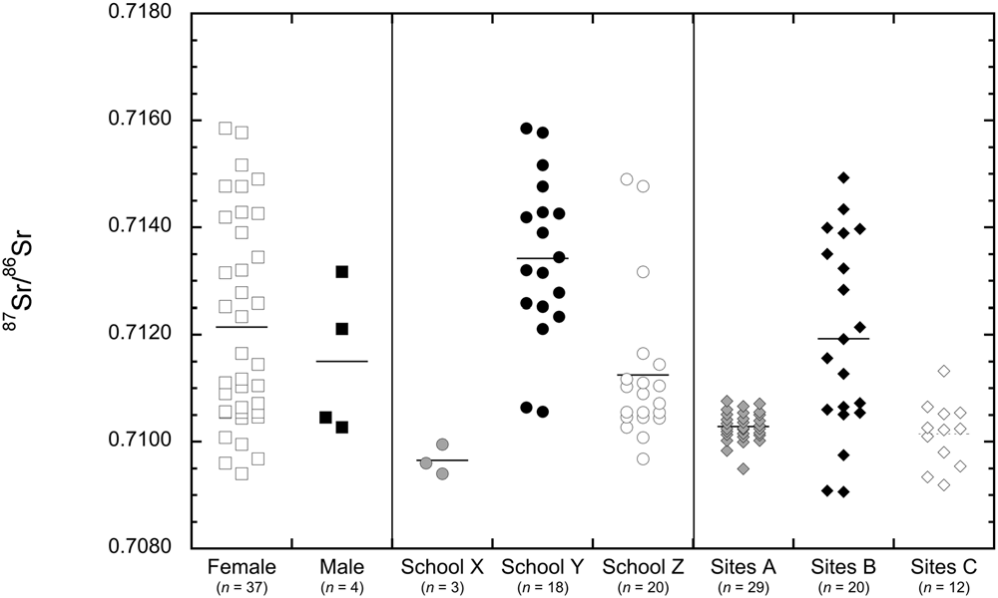
Dot plot showing the ^87^Sr/^86^Sr of hair from female and male donors, ^87^Sr/^86^Sr of hair from each collection location, and ^87^Sr/^86^Sr of tap water from the three tap water Site Groupings. The line represents the mean ^87^Sr/^86^Sr ratio.

Forty-one hair samples were measured for ^87^Sr/^86^Sr and ranged from 0.70910 to 0.71509 (Fig. 4). The mean ^87^Sr/^86^Sr of hair samples from female and male participants was 0.71215 ± 0.00193 (1*σ*, *n* = 37) and 0.71150 ± 0.00139 (1*σ*, *n* = 4), respectively. Here, we observed the ^87^Sr/^86^Sr of hair from female and male students were not significantly different (Student’s *t-*test, *p* = 0.5210). The mean ^87^Sr/^86^Sr of hair samples from students at Schools X, Y, and Z were 0.70965 ± 0.00028 (1*σ*, *n* = 3), 0.71342 ± 0.00152 (1*σ*, *n* = 18), and 0.71125 ± 0.00142 (1*σ*, *n* = 20), respectively. These differences in ^87^Sr/^86^Sr of hair samples at individual schools were significantly different from one another (one-way ANOVA, *p* < 0.0001).

### Strontium isotope ratios of water

Sixty-one tap water samples from six collection sites were analyzed for ^87^Sr/^86^Sr; they ranged from 0.70906 to 0.71399 (Table 1). Tap water samples were grouped based on proximity to hair sampling locations into Site Groups. The mean ^87^Sr/^86^Sr of tap water samples from Sites 1, 2, and 3 (Site Group A), Sites 4 and 5 (Site Group B), and Site 6 (Site Group C) were 0.71028 ± 0.00028 (1*σ*, *n* = 29), 0.71192 ± 0.00181 (1*σ*, *n* = 20), and 0.71014 ± 0.00060 (1*σ*, *n* = 12), respectively (Fig. 4). We found the mean ^87^Sr/^86^Sr of Site Groups were significantly different from one another (one-way ANOVA, *p* < 0.0001).

## Discussion

Oxygen isotope analysis of human hair is becoming a relatively common tool in determining the region-of-origin of modern humans^3,5,38^. Here, we observed the *δ*^18^O of hair from individuals to be indistinguishable at the sites sampled in Salt Lake City, Utah, likely because the origins of urban tap waters share a common montane origin^29,39^. These data are consistent with previous reports of the *δ*^18^O of hair from humans and animals living in or near this region^1,27^. Previously, Ehleringer, *et al*.^1^ found that the average *δ*^18^O of human hair from individuals living in Utah was 9.7 ± 1.5‰ (1*σ*, *n* = 12). When compared to our dataset, we found no significant difference between our data and these previous data (Student’s *t-*test, *p* = 0.6478). The *δ*^18^O of hair has been linked to the *δ*^18^O of drinking water in humans^40–42^ and to assess the relationship between individuals and their drinking water, we translated the *δ*^18^O of hair to *δ*^18^O of drinking water following Ehleringer, *et al*.^1^. Consistent with expectations, the *δ*^18^O of drinking water predicted from measured *δ*^18^O of hair was not significantly different than measured *δ*^18^O of tap water. This is in agreement with previous studies that indicate the *δ*^18^O of an individual’s hair largely relates to the *δ*^18^O of their drinking water, and thus the environment and community in that they reside^3,43–45^. Further, it is not unexpected that there were no differences in the *δ*^18^O of hair from these individuals (Figs 2 and 3), given the relatively small range of *δ*^18^O of tap water within the regions where these individuals were residing.

Studies of the *δ*^18^O of tap water have described spatial and temporal dynamics within single metropolitan areas, including in those around Salt Lake City^28–30^. These dynamics may confound the application of *δ*^18^O of human hair as a geographic predictor, particularly within regions that utilize transported water^46,47^. Within the Salt Lake metropolitan area, Jameel *et al*.^29^ showed that different regions within the study area utilized different management strategies to supply culinary water, resulting in subtle, but unique *δ*^18^O values of tap water across the area. During the three studied seasons, Jameel *et al*.^29^ found the *δ*^18^O of tap water delivered to Salt Lake City had a relatively small range of isotope values (i.e., −16.5 to −14.0‰), consistent with our findings. This was not unexpected as all waters delivered to Salt Lake City ultimately originated from cold season, high elevation precipitation^29,39^. However, the utility supplying Salt Lake City utilized both surficial and groundwater sources depending on season and supply level^48^. While surficial and groundwater sources would have similar *δ*^18^O values given their common source, other geochemical tracers likely vary due to water-rock interactions and other processing. If there are underlying geographic controls of the supply of groundwater to an urban region (e.g., proximity to groundwater wells, etc.), then additional geochemical or isotopic tracers may be useful to uncover intra-city scale variations in water supply and possibly chemical signatures within the individuals residing within these regions.

Strontium in human tissue is generally considered a conservative tracer of geographic origin^22^, however, in human hair the [Sr] and ^87^Sr/^86^Sr are controlled by both diet and environmental contamination^33,34^. The endogenous and exogenous Sr pools in human hair may have unique or quite similar ^87^Sr/^86^Sr depending on the Sr source and either of these pools may or may not preferentially influence the final Sr isotope value^34,49^. Multiple methods have been developed to potentially isolate endogenous and exogenous Sr for dietary and provenancing studies in humans, yet no consensus has been reached on the preferred methodology^34,36,50–56^. Regardless of these known uncertainties, the ^87^Sr/^86^Sr of human and animal hair have been shown to trace movement histories^27,37,57^. However, it remains unclear if the geographic signal within ^87^Sr/^86^Sr in hair relates to the endogenous or exogenous Sr signature, or some combination of both.

Here, we found the range of [Sr] in hair was in good agreement with previous studies of hair from students^58,59^. We observed [Sr] of hair from female participants was significantly higher than [Sr] of hair from males. This too was consistent with other studies that have found that [Sr] in hair a key element to differentiate females and males^58^. In previous studies, [Sr] has been linked to sex, with females most often having greater [Sr] than males^58–63^. While the reason(s) behind this pattern remains unclear, it has been suggested that differences in physiology, biochemistry, diet, and/or activity levels may be the causes of these patterns^31,33,64^. [Sr] is known to increase along the length of hair from proximal to distal portions due to the incorporation of exogenous strontium on and/or into the hair cuticle^27,35,65^. Given that our dataset is weighed towards female participants and females are more likely to wear their hair longer than males, these differences may be related to female participants’ hair incorporating exogenous strontium for extended periods of time, and thereby increasing the [Sr].

While we observed a difference between [Sr] of hair from females and males, we did not find any significant differences in [Sr] of hair samples from collection locations within Salt Lake City. Given the small geographic area from that the individuals resided and our dataset bias towards female participants, it may not be unexpected that there were no differences in [Sr] of hair samples between the three collection locations.

In contrast to [Sr] and *δ*^18^O values of hair, we observed an unexpectedly wide range in the ^87^Sr/^86^Sr of hair. Here, we found the ^87^Sr/^86^Sr of hair from individuals residing in Salt Lake City varied by more than 0.005. This variation was larger than any previously reported ^87^Sr/^86^Sr from hair specimens from individuals residing in the same city or geographic region^34,37^. In a previous study using hair specimens collected from a single salon in Taylorsville, Utah located less than 10 km south of Salt Lake City (Fig. 1), Tipple and colleagues^34^ found the ^87^Sr/^86^Sr of hair ranged from 0.70909 to 0.71469 with an average of 0.71203 ± 0.00140 (1*σ*, *n* = 22)^34^. Our data were not statistically different than these previous data from Taylorsville, Utah (Student’s *t*-test; *p* = 0.9050). Given the constrained geographic area with identical climate and underlying geology, these variations point towards an additional control for the wide range of ^87^Sr/^86^Sr of hair observed within this single metropolitan area.

There remains no consensus regarding the dominant geospatial control on the ^87^Sr/^86^Sr of hair^27,34,37,57^. Several studies have assessed the relationship between the ^87^Sr/^86^Sr of modern hair and residence with some studies suggesting that the endogenous signal to hair is most important for geospatial relationships^57^, while others found compelling relationships with exogenous sources, such as water, atmospheric dust, etc.^27,37^. Unraveling the significant contributions of Sr to hair for geospatial reconstructions is becoming more important in both modern cold case^66^ and archeological^67,68^ contexts.

We found the overall range of ^87^Sr/^86^Sr of hair and tap water was similar at the locations sampled in Salt Lake City (Fig. 4). Yet, the ^87^Sr/^86^Sr of hair from participants varied systematically by collection location within the city (Fig. 4). We noted that participants from School Y had higher ^87^Sr/^86^Sr ratios and a much larger range than the other two collection locations. Previously, Vautour and colleagues^37^ noted that four individuals that traveled from Paris or St. Benoit sur Loire, France to Montreal, Canada showed a transition in ^87^Sr/^86^Sr along a length of hair corresponding to the change in location. They found that the ^87^Sr/^86^Sr of hair from these individuals converged towards an average value with some slight differences between the four individuals and used this finding as evidence that the Sr in hair is not only controlled by endogenous inputs, but exogenous contributions as well. Furthermore, they noted that the ^87^Sr/^86^Sr of hair was similar, but distinct from the ^87^Sr/^86^Sr of local tap water and argued that this was due to reworking and buffering of the Sr isotopic signal from Sr incorporated within bone^37^. Similarly, Font and associates^57^ argued that the ^87^Sr/^86^Sr of hair from two individuals that traveled from Kanpur, India to Amsterdam, The Netherlands slowly reached isotopic equilibrium with the new location due to recycling and incorporation of Sr from bone^32^. While both dietary Sr and remobilization of bone Sr likely contribute to overall ^87^Sr/^86^Sr of hair, the impact of the ^87^Sr/^86^Sr of water on hair may not have been fully appreciated in these previous studies. Here, we analyzed sixty-one water samples in a single municipality and found an astonishingly wide range of ^87^Sr/^86^Sr ratios. These previous studies analyzed five or fewer tap waters from each of the locations that the individuals resided, and thus, may have not captured all the possible ^87^Sr/^86^Sr variability that could exist. Here, we found spatial relationships between the ^87^Sr/^86^Sr of hair and tap water from the neighborhoods study participants resided in, suggesting interactions with local tap waters may contribute to the ^87^Sr/^86^Sr of hair. While the specific mechanism Sr uptake remains unclear, it may take place through imbibing local tap waters or when the hair is wetted during bathing or showering. While this study cannot specifically distinguish between the incorporation of endogenous or exogenous Sr through interactions with water, previous research has shown that significant amounts of Sr can be added to hair after it exits the scalp and is exposed to the environment^33^. Thus, we hypothesize that interactions with bathing water may be an important contributor of Sr to hair and that small spatial variations in the ^87^Sr/^86^Sr of water are possibly recorded in the ^87^Sr/^86^Sr of hair. Geospatial modeling of bioavailable ^87^Sr/^86^Sr for human provenancing remains nascent^19–21,69–71^ and our results suggest that these modeling exercises should consider additional factors or layers for applications towards modern humans^72^, including the ^87^Sr/^86^Sr of tap waters and the processes by which communities acquire and transport their water resources. Nonetheless, additional controlled dietary studies that consider the ^87^Sr/^86^Sr of food, beverages, and external Sr sources will be required to test this hypothesis and establish a mechanism for Sr incorporation from bathing water.

## Methods

### Ethics statement

The Institutional Review Board (IRB) of the University of Utah approved this research program under protocol number [00035524]. Specifically, all sampling and analytical methods used were in accordance with these IRB regulations. Informed consent was obtained from all subjects or from their legal guardians in accordance with and maintained under IRB regulations.

### Hair and water samples

Sixty-seven hair samples were obtained from students from three public schools following Valenzuela, *et al*.^73^ (Fig. 1). Schools X and Y are public schools that pull students from defined areas near the school, while School Z is a public magnet school that has students from throughout the public school district. All study participants lived in Salt Lake City, Utah. Participants were assigned a randomized sample identifier and all personal and identifying information was stored according to IRB. Hair samples were placed in paper coin envelopes and labeled with sample identifier. Hair samples were returned to laboratory and stored. In addition to hair samples, participants provided their sex, along with any specific dietary or health information.

Water samples were collected from taps in six public buildings four times a year for four years (summer, 2012 to summer, 2015). Water collection sites were located within a few blocks of hair collection locations (Fig. 1). The plumbing was flushed prior to sample collection by running the cold-water tap for 10 s. Water samples were collected in 4-ml glass vials and the cap seal was with Parafilm^**®**^. Water samples were returned to laboratory and stored in 4 **°**C refrigerator prior to analysis.

### Hair cleaning

Hair samples were cleaned with 2:1 chloroform:methanol (v/v). Samples were enclosed in filter paper and completely submerged in the solvents. Samples were agitated using a shaker plate and after 5 min the supernatant was decanted and discarded. This process was repeated a total of three times. Chloroform (OmniSolv^®^, EMD; Darmstadt, Germany) and methanol (OmniSolv^®^, EMD; Darmstadt, Germany) used in hair cleaning were HPLC grade. Cleaned hair samples were allowed to dry at room temperature for 72 hrs within a laminar flow hood. After dried, cleaned hair samples were ground in a ball mill and stored in ashed glass vials until further analysis-specific preparation procedures.

### Oxygen isotope analysis of hair

Hair samples equilibrated with ambient laboratory atmospheric water vapor for 48 hrs alongside in-house keratin reference materials (RMs) following Chau, *et al*.^27^. Hair samples (~150 mg), as well as RMs, were weighed in 3.5 × 5 mm silver capsules (Costech Analytical Technologies, Inc.; Valencia, California, USA). Weighed samples and RMs were stored under vacuum for 7 days before being analyzed. The stable oxygen isotope values of keratin were determined with a continuous flow isotope ratio mass spectrometer (IRMS) (MAT 253, Thermo Finnigan; Bremen, Germany), housed at IsoForensics, Inc. in Salt Lake City, Utah. Samples were introduced to the IRMS via a zero-blank autosampler attached to a high temperature conversion elemental analyzer (TC/EA, ThermoFinnigan; Breman, Germany). Samples were analyzed alongside sets of natural keratin RM for primary quality assurance and secondary quality control (QA/QC). Keratin RMs were previously calibrated to Vienna Standard Mean Ocean Water (VSMOW)-Standard Light Antarctic Precipitation (SLAP) international isotope scale. Sets included two primary QA RMs for slope/intercept normalization and a secondary QC RMs to insure suitable calibration. Primary keratin QA RMs were DS and ORX (*δ*^18^O = 6.02‰ and 25.09‰, respectively), while POW was used for QC (long-term mean *δ*^18^O = 12.44‰, 1*σ* = 0.54‰, *n* = 335). A minimum of four sets of RMs was analyzed along side samples. All *δ*^18^O values of hair are expressed relative to VSMOW. All light stable isotope values are reported in parts per thousand (‰) and in delta notation:

$$\delta =[({R}_{samp}/{R}_{std})-1],$$

where *R* represents the ^18^O/^16^O abundance ratio, and *R*_*samp*_ and *R*_*std*_ are the ratios in the sample and standard, respectively.

### Oxygen isotope analysis of water

Water samples were analyzed using cavity ring-down water isotope spectroscopy (L1102-I, Picarro; Sunnyvale, California). Each sample was analyzed four times (four consecutive replicate injections) alongside a set of three liquid water laboratory RMs, (ZE = −0.2‰, EV = −10.2‰, DI = −16.5‰ for *δ*^18^O values) that had previously been calibrated to the VSMOW-SLAP international isotope scale. Two QA RMs were used for data normalization (ZE and DI) and a QC RM for quality control (EV). Analytical precision of the QA RM was ± 0.3‰ for *δ*^18^O values. All *δ*^18^O values of water are expressed relative to VSMOW.

### Digestion for Sr abundance and isotope analysis

Hair samples were digested using an Ethos EZ^®^ microwave digestion system (Milestone, Inc.; Shelton, Connecticut, USA). Approximately 50 mg of hair was weighed into a Teflon^®^ digestion microvessel. Two milliliters of concentrated ultrapure concentrated HNO_3_ (Aristar^®^ ULTRA, BDH Chemical; Darmstadt, Germany) were added to the microvessel containing the hair and the microvessel was then sealed, submerged in 10 ml of milli-Q water and 50 ml H_2_O_2_ (30% v/v), and then placed within an outer vessel. The outer vessel was then placed in the digester carousel. Two certified reference materials (TORT-2, National Research Council, Ottawa, Canada; Human Hair No. 13, National Institute for Environmental Studies, Tsukuba, Japan) and a method blank of reagents were digested along with the hair samples. The microwave program used for hair digestion was 13.3 °C/min ramp to 200 °C, followed by an isothermal at 200 °C for 15 min with a 60 min cool down to room temperature. The microwave was operated at full power (1500 W) for all heating cycles. Once cooled to room temperature, the hair digests were transferred to acid-leached 2-ml snap-cap centrifuge tubes. A 100-ml aliquot of the primary hair digest was transferred to a 15-ml tube and the volume was brought to 10 ml with ultrapure water. The ultrapure water used for sample cleaning and acid dilutions was from a Milli-Q Academic A10^®^ system (EMD Millipore; Billerica, Massachusetts, USA) with a resistivity >18 MΩ. A standard solution containing 10 ppb In was added to each sample as an internal concentration standard.

### Strontium abundance analysis

All strontium elemental abundances were measured via inductively coupled plasma quadrupole-mass spectrometry (ICP-MS) (Agilent 7500ce, Agilent Technologies; Santa Clara, California, USA) at the Department of Geology & Geophysics at the University of Utah, Salt Lake City, Utah. A double-pass spray chamber with perfluoroalcoxy fluorocarbon (PFA) nebulizer (0.1 mL/min), a quartz torch, and nickel cones were used. A calibration solution containing Sr was prepared gravimetrically using a single-element standard (Inorganic Ventures, Inc.; Christiansburg, Virginia, USA). Standard reference solution T-205 (USGS; Reston, Virginia, USA) was measured as an external calibration standard at least five times within each analytical run. The long-term reproducibility for T-205 and differences relative to the accepted values indicated that the Sr concentrations were accurate within 10%. TORT-2 has a certified Sr concentration of 45.2 ± 1.9 mg g^−1^ (1*σ*) and the measured Sr concentration of TORT-2 was 41.2 ± 4.4 mg g^−1^ (1*σ*, *n* = 6).

### Strontium isotope analysis

All strontium isotope measurements were made using a Neptune *Plus* multi-collector ICP-MS (Thermo Fisher Scientific; Bremen, Germany) housed in the Department of Geology & Geophysics at the University of Utah, Salt Lake City, Utah, USA. Digests were introduced using an online Sr purification method following Tipple, *et al*.^34^ for hair samples and Chesson, *et al*.^74^ for water samples. This online system automates the purification of Sr by utilizing a peristaltic pump, a pair of 6-way valves, an in-line separation column, and a SC-2 DX autosampler with a FAST2 valve block (Elemental Scientific; Omaha, Nebraska, USA). The in-line separation column was packed with crown ether Sr resin (Eichrom Technologies; Lisle, Illinois, USA). Variable speed settings on the peristaltic pump allowed samples to be rapidly loaded into the purification column where Sr was trapped while all other elements were rinsed away; the column flow was then reversed and purified Sr was eluted into the spray chamber. A timing solution containing 66 ppb Sr was analyzed daily to insure proper chromatography and to assess column chemistry and efficiency. The instrument was operated at an RF power of 1200 W with nickel sampling and skimmer cones (1.1 mm and 0.8 mm apertures, respectively) and was optimized daily for signal intensity and stability. Cool, auxiliary and sample gas flow rates were 16 L/min, 0.85 L/min, and 0.91 L/min, respectively. The instrument was tuned for sensitivity daily with a solution containing 20 ppb Sr. For ^87^Sr/^86^Sr analysis, a static multi-collector routine was used that consisted of 1 block of 170 cycles with an integration time of 1.032 sec per cycle for an individual analysis. Each analysis was followed by a blank to monitor the efficiency of the crown ether Sr resin column. Sr isotope ratios of samples and references were blank- and interference-corrected and then normalized for instrumental mass discrimination using a defined ^86^Sr/^88^Sr of 0.1194.

Samples were analyzed for ^87^Sr/^86^Sr alongside sets of reference materials and blanks. Reference sets included one primary QA reference for normalization and a keratin QC references to insure measurement reproducibility. The primary reference material was SRM^**®**^ 987 (0.71034 ± 0.00026 [95% CI]; National Institute of Standards and Technology; Gaithersburg, Maryland, USA), while Human Hair No. 13 was used for QC. Samples and SRM^**®**^ 987 were analyzed at 5:1. The measured ^87^Sr/^86^Sr of SRM 987 and Human Hair No. 13 was 0.71030 ± 0.00004 (1*σ*, *n* = 92) and 0.70827 ± 0.00004 (1*σ*, *n* = 6), respectively.

### Statistical analysis

Statistical analysis was completed using JMP Pro 13^®^ (SAS Institute Inc.; Cary, NC, USA). Normality of the distributions were tested with the Shapiro-Wilk test. If the distributions were normal, then the Student’s *t*-test was used to compare means at *α* = 0.005. If the distributions were not normally distributed, then the Welch’s *t*-test was used at differences of *α* = 0.005. One-way ANOVA test was used to assess differences between groups at *α* = 0.005.

### Mapping

Mapping and original figure creation was conducted using ArcGIS 10.4 (ESRI; Redlands, CA, USA).

### Tap water sources

The culinary water delivered to Salt Lake City originates from mountain streams, surface water reservoirs, and groundwater wells and springs^48^ with each source having a distinct ^87^Sr/^86^Sr value (Supplemental Data Table S1). Groundwater in the Salt Lake Valley has more a radiogenic ^87^Sr/^86^Sr than surface water sources (Supplemental Data Table S1). School Y is located near several groundwater wells with groundwater being most likely utilized in the autumn and winter (*personal communication*, M. Hubbard-Rice, Salt Lake City Public Utilities).

## Electronic supplementary material

Supplementary Dataset 1
